# Calcified Leiomyoma of the Distal Forearm in a Child: A Case Report and Review of Literature

**DOI:** 10.1155/2020/8821265

**Published:** 2020-08-28

**Authors:** Nadeeke Nidhan Perera, Sunil Ranjith Wijayasinghe, Karuna Dissanayake, Jerard Fernando, Ethige Tharushi De Silva

**Affiliations:** ^1^National Hospital of Sri Lanka, Colombo, Sri Lanka; ^2^Lady Ridgeway Hospital for Children, Colombo, Sri Lanka; ^3^Colombo North Teaching Hospital, Ragama, Sri Lanka

## Abstract

**Background:**

Deep somatic leiomyomas arising in skeletal muscle are extremely rare in children, especially in the extremities. Around half of them show calcifications. We present a rare case of a calcified leiomyoma of the distal forearm in a child. *Case Summary*. A seven-year-old boy presented with right distal forearm and wrist pain with restricted supination for 4 years. X-ray showed ring and arc calcifications in the distal forearm at the interosseous area. MRI also confirmed a well-defined soft tissue lesion with areas of calcifications. A diagnosis of a cartilage-forming lesion or a peripheral nerve sheath tumour was suggested. The lesion was completely excised. Histology showed a lesion composed of intersecting fascicles of spindle cells with stromal calcification having immunohistochemical features of a leiomyoma.

**Conclusion:**

Although soft tissue calcifications can be seen in a plethora of conditions seen in daily orthopaedic practise, a high index of suspicion should be maintained for rare conditions like deep somatic leiomyoma.

## 1. Introduction

Leiomyomas are benign soft tissue tumours rarely seen in the extremities. Representing 4.4% of all soft tissue tumours, they maybe calcified in around half of the cases [1]. It is also unusual to find them in children. When present in the extremities, it is most likely to be seen in the lower limbs rather than in the upper limbs [2]. Due to the calcification, the radiological features can be misleading and may be misdiagnosed for more commonly seen familiar musculoskeletal conditions. We present an unusual case of a calcified leiomyoma in the distal forearm of a child. This case report is presented according to the CARE guidelines [3].

## 2. Case Presentation

**Figure 1:**
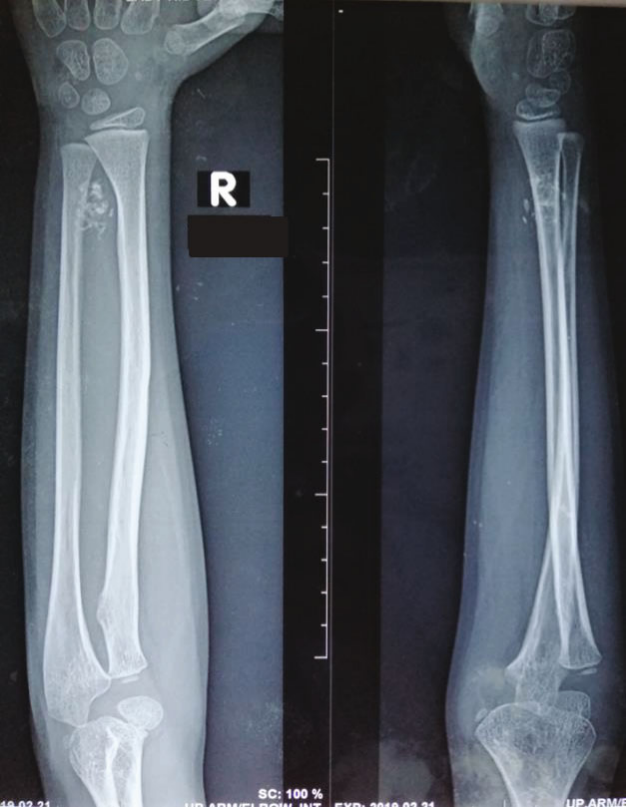
Anteroposterior and lateral views of the right forearm demonstrating ring and arc calcifications in the distal forearm at the interosseous area in close proximity to the metadiaphysial region of the ulna.

**Figure 2:**
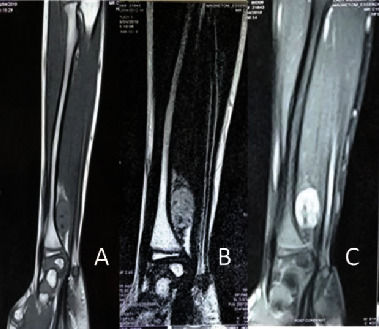
MRI showing a well-defined contrast-enhanced image of a soft tissue lesion in the forearm. (a) T1W: hypointense. (b) T2W/STIR: hyperintense. (c) Post contrast.

A seven-year-old Sri Lankan boy presented with a gradually worsening right distal forearm and wrist (dominant side) pain for four years. He was previously well and had no constitutional symptoms. He also denied having any numbness or tingling sensation in the involved area of the upper limb. The pain and functional limitation were interfering with his daily activities and schooling. There was no family history of malignancies. There were no obvious lumps noticed by the child or the parents. He was from a middle-income family living in the Colombo suburbs and was the only child. The cause of the child's pain had not been thoroughly investigated previously. On examination, there was no obvious lump or overlying skin changes detected. There was moderate tenderness over the distal forearm. Pronation was normal (0-90°), while supination was restricted (0-20°). Neurovascular examination of the right hand was normal. All blood investigations and inflammatory markers were within normal limits. Plain X-ray showed ring and arc type calcifications in the distal forearm at the interosseous area, in close proximity to the metadiaphyseal region of the ulna (Figure 1). The medial aspect of the distal radius showed remodelling due to possible longstanding pressure effects. There was no cortical destruction or periosteal reaction. Based on these X-ray findings, a diagnosis of a benign chondroid lesion was initially suggested. A soft tissue chondroma or a low-grade malignant chondrosarcoma was considered; however, a periosteal chondrosarcoma was also within the realm of possibilities. MRI confirmed the well-defined soft tissue lesion deep in the forearm muscles with areas of signal voids consistent with the calcifications. The lesion was hypointense in T1W and hyperintense in T2W/STIR images with postcontrast enhancement. A diagnosis of a peripheral nerve sheath tumour was also suggested based on the MRI (Figure 2).

**Figure 3:**
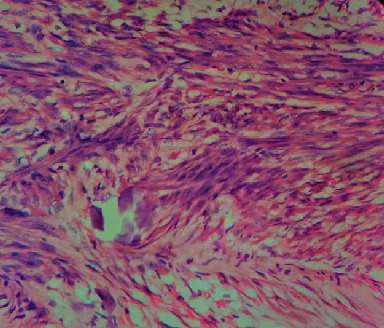
Histology showing intersecting fascicles of spindle cells with elongated nuclei and eosinophilic cytoplasm. Stromal calcification is also seen (H&E stain, ×100).

Surgery was performed under a bloodless field via the trans-FCR approach [4]. The lesion was found to be deep in the pronator quadratus with the muscle adhering to the lesion as a thin sheath. A mass measuring 2.5 cm × 1.5 cm was completely excised by enucleation. It was oval, well defined, and rubbery in consistency. Histology showed a circumscribed unencapsulated lesion composed of intersecting fascicles of spindle cells with elongated nuclei and eosinophilic cytoplasm in the H&E stain (Figure 3). Nuclear atypia was not seen. Mitotic figures were noted occasionally (<1/10 hpf). Tumour necrosis was not evident. Foci of stromal calcifications were present appearing fractured in nature. Tumour was present in the inked margin. Immunohistochemistry studies were positive for desmin and smooth muscle actin (SMA) but negative for bcl-2, epithelial membrane antigen (EMA), MyoD1, and S-100. A diagnosis of leiomyoma in the deep soft tissue with psammomatous calcification was made. The recovery was uneventful at six months.

## 3. Discussion

Leiomyoma is a benign tumour of smooth muscle origin usually occurring in the areas of the body composed of smooth muscles, such as the uterus or the gastrointestinal tract. When seen in soft tissues, it usually involves the dermis and subcutis, and very rarely, the deep soft tissue. It is classified as cutaneous leiomyoma (leiomyoma cutis), angiomyoma (vascular leiomyoma), or leiomyoma of deep soft tissue [5]. Historically, the presence of deep soft tissue leiomyoma as a separate clinical entity was doubted. However in 2001, Billings et al. published a series of 36 cases where the authors established the histological subtypes of soft tissue leiomyoma and now it is widely accepted to be a rare tumour. Leiomyoma of the deep soft tissues are exceptionally rare compared to its malignant counterpart. Therefore, strict diagnostic criteria must be employed to establish the diagnosis [1]. Mostly arising from the skeletal muscles of the extremities, they are thought to originate from vessels where smooth muscle tissues can be found. Due to its scarcity, much of our knowledge regarding this entity is based on case reports and small case series. They are very rarely described in children and are more common in the adult population. This tumour usually shows calcification in 58% of the cases [6]. Leiomyomas in the skeletal muscles have been described in 12 children so far in case reports and case series [1, 2, 6–11] including our case report (Table 1). The only instances where such tumours were reported in the forearm of an adult is of a 25-year-old female [12].

The significance of our case lies in its rarity and the diagnostic dilemma it entailed due to the nonspecific radiological appearance. Calcified appearance in soft tissue may give rise to diagnostic confusion and may point towards familiar musculoskeletal conditions. The ring and arc calcifications in our case initially pointed towards a benign or malignant cartilage-forming pathology. Among other conditions that can be considered are calcified haematoma, myositis ossificans, tumoural calcinosis, tuberculous lymphadenitis, pilomatrixoma, calcifying fibrous pseudotumour, synovial sarcoma, mesenchymal chondrosarcoma, and extraskeletal osteosarcoma [2, 9, 13].

Histologically, these are composed of mature-appearing smooth muscle cells with abundant eosinophilic cytoplasm. In order to diagnose soft tissue leiomyomas, lesions should have no cytological atypia and no coagulative necrosis. They should also be minimal to amitotic (<1 mitoses/50 hpf) [14]. Immunohistochemistry (IHC) is helpful when diagnosis is doubtful. Leiomyomas of deep somatic soft tissues are positive for SMA, desmin, and caldesmon [15]. Bcl-2 and EMA were done to exclude synovial sarcoma, while MyoD1 was helpful in excluding the possibility of rhabdomyosarcoma. S-100 was done in our case to exclude a peripheral nerve sheath tumour [5] as it was suggested based on MRI appearance albeit being rare in this age group [16]. Wide local excision is the treatment of choice in these lesions. Even in the presence of positive margins, these tumours are not reported to recur or metastasise [1].

## 4. Conclusion

Deep somatic leiomyomas arising from skeletal muscle are extremely rare in children, especially in the extremities. A calcified leiomyoma of the distal forearm in a child has not been previously recorded in published literature. It should be considered in the differential diagnosis of any extensively calcified deep soft tissue lesion in children. Histopathological/immunohistochemical confirmation is needed to exclude its more common malignant counterpart.

## Figures and Tables

**Table 1:** Details of the published cases of deep soft tissue leiomyoma of the extremities in children.

Author and year	Case no	Age	Sex	Pain	Duration of symptoms	Site	Calcification	Size
Goldman (1964)	1	5 yrs	Female	No	5 months	Thigh	Yes	7.5 cm
Bulmer (1967)	2	7 yrs	Male	No	3 weeks	Arm	No	3 cm
Ledesma-Medina (1980)	3	6 yrs	Male	Yes	n/a	Axilla	Yes	5 × 4.5 × 3 cm
	4	3 yrs	Male	n/a	6 weeks	Subscapular	Yes	8 × 7 × 3.2 cm
Ross (1983)	5	11 yrs	Male	Yes	Few weeks	Calf	Yes	7 cm
Lopez-Barea (1994)	6	6 yrs	Male	Yes	6 months	Calf	Yes	5.5 × 3.5 × 3 cm
Billings (2001)	7	12 yrs	Male	n/a	n/a	Thigh	n/a	n/a
	8	14 yrs	Female	n/a	n/a	Forearm	n/a	<1 cm
	9	6 yrs	Male	n/a	n/a	Thigh	n/a	2 cm
Rasool (2013)	10	3 yrs	Male	Yes	6 months	Calf	Yes	9 × 5 cm
Ramachandran (2014)	11	16 yrs	Male	Yes	2 months	Forearm	No	n/a
Present case	12	7 yrs	Male	Yes	4 yrs	Distal forearm	Yes	2.5 × 1.5 cm

n/a: not available.

## Data Availability

The clinical details, investigation reports, and radiological and histopathological details used to support the findings of this study are available from the corresponding author upon request.
